# Strategies and methods to study sex differences in cardiovascular structure and function: a guide for basic scientists

**DOI:** 10.1186/2042-6410-2-14

**Published:** 2011-12-12

**Authors:** Virginia M Miller, Jay R Kaplan, Nicholas J Schork, Pamela Ouyang, Sarah L Berga, Nanette K Wenger, Leslee J Shaw, R Clinton Webb, Monica Mallampalli, Meir Steiner, Doris A Taylor, C Noel Bairey Merz, Jane F Reckelhoff

**Affiliations:** 1 Departments of Surgery, Physiology and Biomedical Engineering, Mayo Clinic, 200 First Street SW, Rochester, MN, 55905, USA; 2 Department of Pathology (Comparative Medicine), Wake Forest School of Medicine, Winston-Salem, NC, USA; 3 The Scripps Research Institute and The Scripps Translational Science Institute, La Jolla, CA, USA; 4 Department of Medicine, Johns Hopkins University, Baltimore, MD, USA; 5 Department of Obstetrics and Gynecology, Wake Forest University, Winston-Salem, NC, USA; 6 Department of Medicine, Division of Cardiology, Emory University School of Medicine, Atlanta, GA, USA; 7 Emory Clinical Cardiovascular Research Institute, Emory University School of Medicine, Atlanta, GA, USA; 8 Department of Physiology, Georgia Health Sciences University, Augusta, GA, USA; 9 Science Department, Society for Women's Health Research, Washington, DC, USA; 10 St Joseph's Healthcare, Hamilton, Ontario, Canada; 11 Department of Integrative Biology and Physiology, Center for Cardiovascular Repair, University of Minnesota, Minneapolis, MN, USA; 12 Women's Heart Center, Cedars-Sinai Heart Institute, Los Angeles, CA, USA; 13 Women's Health Research Center and Department of Physiology and Biophysics University of Mississippi Medical Center, Jackson, MS, USA

**Keywords:** animal models, estrogen, gender, genomics, sex chromosomes, sex steroid hormones, study design, testosterone

## Abstract

**Background:**

Cardiovascular disease remains the primary cause of death worldwide. In the US, deaths due to cardiovascular disease for women exceed those of men. While cultural and psychosocial factors such as education, economic status, marital status and access to healthcare contribute to sex differences in adverse outcomes, physiological and molecular bases of differences between women and men that contribute to development of cardiovascular disease and response to therapy remain underexplored.

**Methods:**

This article describes concepts, methods and procedures to assist in the design of animal and tissue/cell based studies of sex differences in cardiovascular structure, function and models of disease.

**Results:**

To address knowledge gaps, study designs must incorporate appropriate experimental material including species/strain characteristics, sex and hormonal status. Determining whether a sex difference exists in a trait must take into account the reproductive status and history of the animal including those used for tissue (cell) harvest, such as the presence of gonadal steroids at the time of testing, during development or number of pregnancies. When selecting the type of experimental animal, additional consideration should be given to diet requirements (soy or plant based influencing consumption of phytoestrogen), lifespan, frequency of estrous cycle in females, and ability to investigate developmental or environmental components of disease modulation. Stress imposed by disruption of sleep/wake cycles, patterns of social interaction (or degree of social isolation), or handling may influence adrenal hormones that interact with pathways activated by the sex steroid hormones. Care must be given to selection of hormonal treatment and route of administration.

**Conclusions:**

Accounting for sex in the design and interpretation of studies including pharmacological effects of drugs is essential to increase the foundation of basic knowledge upon which to build translational approaches to prevent, diagnose and treat cardiovascular diseases in humans.

## Introduction

There are significant disparities between women and men in the incidence of, and mortality from, cardiovascular disease [1-4]. Thus, there is a need for improvement in the current preventive, diagnostic and treatment strategies by accounting for sex-specific differences in the etiology and risk factors of cardiovascular disease. In 2001, the Institute of Medicine advocated that a better understanding of differences in human diseases between males and females, with translation of these differences into clinical practice, requires consideration of sex as an important biological variable in design of basic research [5]. Funding agencies in the US, Canada and the European Union require inclusion of women in governmental sponsored research and analyses of outcomes by sex [6-10]. Often, however, implementation of those requirements is not met. Even if the requirement is met, including men and women in clinical studies often does not advance the goal of understanding sex differences as there is no requirement to compare the sexes nor is there a requirement to sufficiently power studies to do so [11-15]. Furthermore, there are no requirements for inclusion of male and female animals in basic, mechanistic, or preclinical studies and often sex is not reported as a critical biological variable in the study design [16-18].

This paper is intended as a guide to help formulate hypothesis-driven studies of cardiovascular function accounting for sex as a biological variable. Design considerations for studies on sex differences of cardiovascular disease will be discussed to extend those which have been developed for studies of brain and behavior [19,20], neuroprotection after stroke [21], and pain and analgesia [22]. Advantages and disadvantages of various cell/tissue culture systems and experimental animals (from transgenic mice to non-human primates) will be reviewed along with methods to differentiate sex-specific effects caused by various factors (for example, sex chromosomes and gonadal hormones). Questions are posed to direct investigators toward optimizing the choice of experimental system to maximize the information gained for translation to human medicine. Application of genetic studies including quantitative trait locus mapping will be evaluated in relationship to cardiovascular phenotypes of different model species. Finally, recommendations are provided for statistical analysis and future research directions. Accounting for sex in the design and interpretation of basic research of cardiovascular structure, function and disease is essential to achieve scientific excellence whether such studies utilize cultured cells, isolated tissues, or experimental animals [23,24] and is essential to increase the foundation of basic knowledge upon which to build translational approaches to medical care of humans [25-28]. Information provided in this paper will help guide the investigator toward that excellence.

### Definitions

Sex, a biological construct, gender, a psychosocial construct, and environment contribute to disparities in cardiovascular disease separately or through interactions. Studies using cells and tissues or experimental animals can be designed to identify these differences and interactions.

#### Terminology related to sex steroid hormones

'Sex', a biological construct, refers to biological differences defined by sex chromosomes (XX, XY) and the presence of functional reproductive organs and sex steroids [5,19,20]. Biological questions require identification of which cells, tissues and organs demonstrate dimorphic structure and function related to cardiovascular disease. 'Gender', a cultural construct, refers to behaviors thought to be directed by specific stimuli, such as olfaction, for example, or by psychosocial expectations that result or accrue on the basis of assigned or perceived sex. Thus, gender can influence biological outcomes. For example, in a society where one sex is devalued relative to the other, the devalued gender may experience increased stress through reduced autonomy or access to nutrition/water/sleep, or in the case of humans, access to medical resources, which may influence development of cardiovascular disease, diagnosis and subsequent treatment. Thus, sex is considered a dichotomous variable; gender is a continuous variable as defined by a range of characteristics that might vary with age, species (animals), or ethnicity (humans), geographical location, education, and culture. Most studies using animals categorized by anatomical features and chromosomes can be described as studies of 'sex differences', with identification of dimorphic cellular, tissue, or organ functions. However, studies using animals can also be designed to answer questions which address psychosocial constructs, such as whether hierarchical social interactions are biologically based and whether they influence biological imprinting, or vice versa, in relationship to cardiovascular disease.

#### What are sex steroid hormones?

As described above, sex is defined by the XX or XY chromosomal complement and the presence of sex organs and sex steroid hormones. Sex steroid hormones fall into three general classes of hormones: androgens, estrogens and progestogens. Intracellular and intranuclear actions of hormones belonging to these classes are mediated through binding of the hormones to specific receptors. Extensive reviews of the types and cellular mechanisms of action of these receptors are available [29-36].

Designing experiments in which one intends to evaluate effects of sex steroids on cardiovascular disease processes require the mention of certain caveats. First, hormones belonging to each of these classes are synthesized naturally in mammals. However, synthetic analogs of the naturally produced hormones are also available commercially. Most natural steroid hormones bind to only one class of intracellular receptor. For example, the naturally occurring estrogen, 17β-estradiol, binds only to estrogen receptors and not to an androgen or progesterone receptor, and naturally occurring progesterone binds only to the progesterone receptor. However, many synthetic steroids bind to multiple classes of steroid receptors. Thus, for example, it is important to distinguish synthetic progestogens from the natural hormone as they may differ in their affinity for progesterone and/or glucocorticoid receptors or their antimineralocorticoid effects [32,37,38]. In addition, certain progestogens may bind to the androgen receptor. These may antagonize actions of endogenous androgens by reversibly binding to the androgen receptor but because these progestogens are 'weak androgens', their binding does not initiate the full cascade of intracellular actions such as translocation to the nucleus and initiation receptor-mediated DNA transcription [39-41]. This issue is of particular importance in evaluating effects of hormonal treatments (hormonally based contraception, oophorectomy and hormonal treatments, including those of selective estrogen receptor modulators (SERMS)) on cardiovascular function and disease.

A second consideration regarding studies utilizing sex steroid hormones is that of tissue production independent of primary sources. Although the primary source of production of androgens is the testes in males and the ovary for production of estrogens and progesterone in females, local steroidogenesis and possible production of steroids from 'precursor' compounds at the cell membrane or within the cytoplasm in non-sex organs may also occur. Thus, steroid exposure may occur on a tissue or cell level that is not reflected by measurement of circulating levels of sex steroids [42-46].

The third consideration regarding the evaluation of hormonal effects in design of studies investigating sex differences is that metabolic products of androgens and estrogens have biologic activity. The androgen, 5-dehydroepiandrosterone (DHEA) is synthesized from cholesterol in the adrenal gland, testes and ovaries. Testosterone, as well as five other androgens, is synthesized from DHEA. Testosterone can be aromatized to 17β-estradiol while the androgen, 5α-dihydrotestosterone (DHT), cannot be aromatized. Therefore, for studies in which the investigator administers an androgen but does not want to risk increasing estradiol synthesis, DHT can be substituted for testosterone. However, DHT is more biologically active because it binds to the androgen receptor with a 15-fold higher affinity than testosterone [31].

The primary estrogen produced by the human ovary is 17β-estradiol, but the term 'estrogen' encompasses metabolites of 17β-estradiol including estrone, estrone sulfate, estriol, ethinyl estradiol, as well as xenoestrogens. Because metabolites of 17β-estradiol are biologically active [47,48], investigators must be specific about the hormone used in their studies as well as the mode of delivery. For example, because of a 'first pass' effect through the liver, oral 17β-estradiol is metabolized to estrone and estrone conjugates prior to distribution in the circulation whereas delivery of 17β-estradiol transdermally, subcutaneously, intramuscularly, or vaginally results in a physiological ratio of estradiol to estrone. In human females with intact ovaries during reproductive years, the ratio of 17β-estradiol to estrone is roughly 1:1 [49]. The adrenal glands also manufacture and secrete estradiol and estrone. Oral products through the 'first pass effect' also increase production of liver proteins, such as sex hormone-binding globulin (SHBG) and clotting factors, with attendant biological consequences.

Terminology is also important. For example, a mixture of conjugated estrogen metabolites derived from urine of pregnant mares (conjugated equine estrogen (CEE)), which contains primarily equilin sulfate and estrone sulfate (and other metabolites of estrogen, as well as androgens) was used in the Women's Health Initiative. Oral administration of this compound was used to determine effects of 'estrogen treatment' for primary prevention of cardiovascular disease in postmenopausal women. Publications from this trial refer to the effects of 'estrogen' but, in fact, are the effects of a mixture of steroid metabolites, and thus the findings may not be applicable to other specific estrogenic products delivered by other routes or to results of studies conducted in experimental animals where 17β-estradiol was administered subcutaneously [50-54].

### Design considerations and choice of experimental models

Choice of an experimental system will affect outcomes and should be considered carefully in design of experiments to evaluate sex differences in cardiovascular disease as many unanswered questions exist regarding whether sex differences in a particular phenotype are due to sex, hormones, or sex × hormone interactions at various life stages.

#### Origins of sex differences

All biological sex differences are initiated by the genes of the sex chromosomes, which are the only genes that are inherited in a sex-specific manner. Differences between XY and XX cells can be attributed to: (1) the presence of Y genes only in male cells, (2) the presence of a higher dose of X genes in XX compared to XY cells with random X-inactivation of one X chromosome in XX cells, (3) mixed maternal and paternal genomic imprint on X genes in XX cells, and/or (4) only maternal imprinting on X genes in XY cells [19]. The SRY gene on the Y chromosome, which determines the development of the testis and the subsequent secretion of male sex hormones, also influences expression of other genes on the autosomal chromosomes. Sex hormones at critical periods in development influence cellular differentiation, which may also be influenced by environmental factors. Another way to frame these interactions is as hormone-independent sexual differentiation, hormone-dependent sexual differentiation and sex-specific hormone actions. Determining whether a trait or condition is a sex difference requires a systematic approach to separate the interactions of sex chromosomes, sex hormones and environment. A logical series of experimental questions that has been proposed for studies in brain and behavior [19] can serve as a guide for investigators in cardiovascular disease research as well. These are: (1) is there a sex difference in the trait/phenotype? (2) Does the sex difference result from the hormonal status of the test system (cells in culture, isolated tissues or animals) at the time of testing? (3) Is the hormonal action specific to or modified by sex? (4) Does the sex difference result from permanent differentiating effects of gonadal hormones? (5) Is the sex difference due to genomic or non-genomic responses to the sex steroids? And (6) does the sex difference result from a sex chromosomal influence on autosomal gene expression?

#### Choice of experimental systems

The choice of an experimental system will depend on the question being asked and the hypothesis to be tested. In contemporary publishing, methodological details including the sex of the research material or animals are often omitted from research papers or relegated to supplementary material. However, it is just these methodological details that allow investigators to reproduce experiments of others and to understand how important variables such as sex or hormonal status may influence outcomes. The Institute for Laboratory Animal Research and their official publication (*ILAR Journal*) is a rich resource for details regarding reproductive, hormonal and developmental information of various animal species used in cardiovascular disease research. Several general considerations are provided below for some of the more common experimental systems employed to address mechanisms of cardiovascular function.

##### Cells in culture and isolated tissues

Cultured vascular endothelial cells, smooth muscle cells and cardiac myocytes (including neonatal cells), as well as isolated blood vessels and hearts, are used in experiments that explore intracellular signaling mechanisms in the development and treatment of cardiovascular disease. While most signaling pathways may be common in cells/tissues derived from female and male animals, it is important to understand which pathways may or may not show a sex difference as gene and protein expressions are influenced by both sex and hormones [55-62]. The same can be said for stem and progenitor cells being cultured for cell-based therapies [63-66]. For example in mice, cells derived from female animals appear to be more effective at both reversing disease and restoring a pre-disease level of cells to bone marrow than male-derived cells [66]. Differences in the efficacy of male and female cells could possibly be attributed to differences in paracrine factors (for example, cytokines) [65]. Comparison of these preclinical vascular data to early human clinical data suggest that sex-based differences in progenitor cell numbers and function seen in mouse models of atherosclerosis and acute myocardial infarction reflect human clinical scenarios [64].

The following points should be addressed in design of experiments using cultured cells, isolated stem and progenitor cells, and isolated tissues: (1) is expression of the receptor, pathway, enzyme, and so on, or the number of isolated progenitor cells affected by the sex or hormonal status of the donor animal? For example, would gene expression/signaling differ depending on whether the donor animal was studied before sexual maturity, was sexually mature (null or multiparous), or was reproductively senescent? (2) Would number of cells, gene expression or phenotype be affected by gonadectomy of the donor either before or after sexual maturity? (3) How does the expression of the pathway of interest or phenotype of progenitor cells change with passage of cells exposed to hormones in culture media using fetal bovine serum? (4) Is the choice of cell/tissue donor appropriate for the mechanism of interest related to human disease in regard to sex, age and hormonal status? (5) For cell-based therapies, given potential sex differences in cell number, phenotype, and potency, should sex mismatched allogeneic cells be considered as a therapeutic option? If so, is the donor appropriate for the mechanism of interest related to human disease in regard to sex, age and hormonal status?

The age/hormonal status of the donor for cell/tissue may not be available depending on the source of the material (that is, abattoir, human material for which medical information/records are not available, some immortalized cell lines). If the sex of the cell/tissue donor is not known, it can be determined by a PCR-based assay to identify specific fragments of the X and Y chromosomes. For example, a multiplex PCR-based method was developed to measure a 475-bp fragment from the ABCD1 gene (X chromosome) and a 231-bp fragment from the SRY gene (Y chromosome) [67]. This method has been refined and extended to a high-throughput automated setting [68].

Sex of neonatal animals (including mice and rats) can be determined by examining the anogenital distance [69]. In larger animals the presence of gonads should be confirmed as some male animals obtained from commercial suppliers (in particular, pigs) may be castrated at birth, and, therefore, studies in these animals would yield cells developed or differentiated under a sex hormone-depleted environment.

As sex differences are influenced both by the sex chromosomes and sex hormones, care must be taken to control the hormonal environment of cultured cells. Some media, in particular, fetal calf serum, contain sex steroid hormones that could influence the pathway/signal of interest. Media can be stripped of hormones by charcoal treatment. When hormones are added to the media, care should be taken to control for the solvent used for lipophilic compounds and to consider that both testosterone and 17β-estradiol may be metabolized by the tissue. Thus, a given concentration of sex steroid added at day 1 of a culture cycle may not be sustained over a long period of time. Alternatively, exposure of cells to hormones may 'imprint' the cell phenotype over several passages [70,71]. As sex steroid hormones initiate rapid actions that do not require gene transcription (non-genomic actions) as well as effects on gene transcription (genomic actions) that represent different temporal sequences, duration of exposure to the hormone of interest is a critical consideration in study design.

For example, prolonged exposure of certain cells to 17β estradiol increases synthesis of endothelial nitric oxide synthase (eNOS) via a genomic mechanism caused by binding of the ligand-bound estrogen receptor α (ERα) to an estrogen response element (ERE) on the eNOS gene [37]. However, estradiol also acutely increases eNOS activity and release of nitric oxide via a non-genomic effect that is due to an increase in intracellular calcium [37]. Data also suggest that there is a plasma membrane-associated, G-protein coupled estrogen receptor (GPR30 or GPER) [41]. However, the specificity of this receptor is controversial since GPR30 also mediates rapid aldosterone-mediated effects on the vasculature [32,33,72]. Androgens also produce genomic effects via androgen response elements (AREs) in genes, but cause acute vasodilation by a non-genomic mechanism that involves activation of calcium activated potassium channels [73,74]. Future studies are necessary to completely understand the genomic and non-genomic effects of sex steroids and the mechanisms responsible for modulating cardiovascular function including whether the chromosomal constitution of the cell modulates responses to steroids via transcription factors, differential expression of protein chaperones, methylation of DNA, and so on [32,33,72].

##### Experimental animals

Several mammals are used in studies of cardiovascular disease. General considerations for the selection of the appropriate species include cost, size, housing requirements, diurnal activity cycle, diet requirements (soy or plant based influencing consumption of phytoestrogen) [75], lifespan, frequency of estrous cycle in females, ability to perform genetic manipulations, ability to investigate developmental and/or environmental components of disease modulation [76]. Stress imposed by disruption of sleep/wake cycles, patterns of social interaction (or degree of social isolation), or handling may influence some parameters (for example, corticosteroids and catecholamines) that interact with pathways activated by the sex-steroid hormones [77,78].

Of the small mammals, rats and mice are used most often to model cardiovascular disease because of their short life span, short estrous cycle and gestation, modest cost of housing, and the ability to perform genetic manipulation in them. Both the strain and sex of the animals are known to influence expression of cardiovascular pathologies [79,80].

Transgenic mice (and some transgenic rats) that contain estrogen and androgen receptor knockouts are commercially available for study. Although preliminary, use of these animals to study the onset and progression of vascular disease has shown that the number of vascular progenitor cells found in bone marrow and blood is decreased in estrogen receptor knockout (ERKO) mice compared to wild type controls. These transgenic animals are especially valuable to study the role of sex steroids and their receptors in cardiovascular disease. While the global ERKO mice are viable and fairly healthy, global androgen receptor knockouts (ARKO) have serious developmental problems [81]. For example, the global ARKO exhibit neutropenia, osteopenia, low levels of serum testosterone, arrested spermatogenesis, and females have a reduced number of pups compared to wild type mice [81]. Therefore, especially for androgen receptor, specific tissue-directed androgen receptor knockouts, using Cre-loxP methods, are preferred [82-85]. Cre-loxP technology allows for knockout or expression of a gene of interest in a specific tissue [86]. Briefly, the site-specific recombinase Cre (cyclization recombination) from the bacteriophage P1 is used to induce recombination between two 34 bp recognition sites (loxP = 'locus of crossing over in P1') inserted into the genome. These loxP sites contain two 13 bp inverted repeats surrounding an 8 bp core sequence that provides directionality. Thus, two transgenic mouse strains are necessary to develop a cell-specific knockout: one strain that has the Cre recombinase expressed under the control of a cell specific promoter, and one that contains the gene of interest (or a critical exon of it) that is flanked by two loxP sites (termed 'floxed'). Following one round of crossbreeding, double transgenic pups containing both the floxed transgene and the Cre transgene are selected by genotyping. In a second round of breeding the double heterozygotes are either inbred or bred to the floxed mouse line. In the F2 generation, heterozygotes for the floxed allele and the Cre transgene are selected since the floxed gene/exon will be excised selectively in the cell types that express Cre recombinase (taken from review in [86]). For the androgen receptor, there have been several Cre/loxP mice developed for reproductive tissues, as well as adipose tissues, skeletal muscle, bone, T cells and B cells, liver and skin, to name a few [82]. The use of cell-specific knockouts of AR thus allows the investigator to specifically study the role of AR in their tissue/cell of choice while minimizing the effects of global changes that would complicate the study and perhaps make interpretation ambiguous. It should be kept in mind that the gene for the androgen receptor resides on the X chromosome. Thus, studies androgen actions in females are complicated by X inactivation which results in mosaic expression of any polymorphisms that reside in this gene or others which have the potential to affect cardiovascular function in females.

One caveat about the use of mice, transgenic or otherwise, is that uncertainties still exist regarding the degree to which cardiovascular phenomena, especially the pathobiology of atherosclerosis in these rodents, can be translated to human beings (see review in [87]). However, Taylor and colleagues [65,66,88] have shown in apoE-/- mice and preliminarily in ERKO mice: (1) the onset and progression of atherosclerosis differs in male and female animals with the onset of disease occurring earlier in males and catching up with aging in females, similar to that in humans; (2) that the composition of the bone marrow in male and female animals differs and changes over time with aging consisting with the onset and progression of disease; (3) that the use of bone marrow mononuclear cells from females is sufficient to decrease atherosclerotic plaque in males but that the converse is not true; and (4) that the number of circulating cytokines differs based on sex and degree of disease with proinflammatory cytokines being higher in the circulations of males than in females.

Based on a spontaneous deletion of the SRY gene from the Y chromosome, Arnold and colleagues have developed transgenic animals for evaluating whether a phenotype follows gonadal sex or sex chromosome complement [89]. For example, these investigators developed transgenic animals to 'knock-in' the SRY gene on an autosome of XX animals to provide mice that have testes but XX chromosomes; alternatively, with the spontaneous deletion of the SRY gene from the Y chromosome, mice have ovaries but XY chromosomes. This allows the investigator to determine if a sex difference in phenotype correlates with the type of sex chromosomes (XX or XY) or with gonadal sex of the animal. Using these intact and gonadectomized animals, investigators can separate the contribution of sex steroids from sex chromosomes (the interaction of sex and sex hormones, that is, sex specific hormone action) in mediating sex differences in cardiovascular function and dysfunction.

In addition to transgenic animals, inbred and congenic strains of rats have been used extensively to study mechanisms responsible for sex differences in cardiovascular disease etiology. For example, Dahl salt sensitive rats exhibit hypertension with aging or more rapidly with a high salt diet [90]. High salt diet-mediated hypertension is exacerbated in males and ovariectomized females and attenuated in castrated males and intact females [91,92]. Spontaneously hypertensive rats (SHR) also exhibit sex differences in blood pressure control, with males exhibiting higher blood pressures than females [93]. Sex differences in the pressor response to angiotensin II have also been documented in mice and rats [94,95].

With regard to rat models that mimic menopause, both SHR and Dahl salt sensitive rats have been used. Both strains of rats exhibit naturally increasing blood pressure when they stop estrous cycling, and have been used to study the mechanisms responsible for postmenopausal hypertension. However, in female rats after cessation of estrous cycling, estradiol levels do not fall as low as in women following menopause which is a common criticism of using rats as a model of menopause. To address this problem, investigators have used 4-vinylcyclohexene diepoxide (VCD), a chemical toxin that causes ovarian failure by targeting pre-antral follicles [96-99]. VCD can be used in rats and mice and allows for studies to be performed during a perimenopausal period [96,100]. Treatment of animals with VCD causes a follicular depleted state and a cessation in the production of female ovarian hormones within approximately 50 to 75 days after injection, with full cessation of estrogen production and obsolescence of the follicles by 129 days [97]. The advantages of this model are twofold. First, the rodents undergo a sustained period with no estrogen production. Second, VCD can be given in a young adult animal to simulate early ovarian failure [101]. Since the VCD ovary produces androgens just as do ovaries of postmenopausal women, perhaps this best models ovarian failure in women [102-106]. The investigator should keep in mind that studies in women with early ovarian failure (19 to 39 years of age) showed that they responded to transdermal estradiol with reductions in blood pressure, angiotensin II and creatinine [107], whereas in women who go through natural menopause (average age 51 years), the effects of estradiol are not as clear which was evident by the results of the Women's Health Initiative [108]. Thus, chemical menopause may be a better option to examine interactions of aging with estrogen depletion rather than the loss of all ovarian hormones as would result from ovariectomy.

There is a growing literature that considers sex differences in mouse strains and cardiovascular disease, sex-specific differences in the impact of particular bone marrow, blood, cardiac and vascular progenitors cells [65,66] and sex-specific influence of particular gene effects [87,109,110]. In this light, many of the strategies used by geneticists to identify and characterize specific gene effects on cardiovascular disease-related phenotypes can easily accommodate tests of hypotheses surrounding the sex specificity of those gene effects, as will be discussed in the section on 'Identifying genes that exhibit sex-specific or sex-interaction effects' below.

Rabbits historically have been used in studies of autonomic and pharmacological regulation of vascular tone and vascular remodeling associated with atherosclerosis, ischemia reperfusion and stroke [111-122]. The Watanabe breed develops spontaneous atherosclerosis [123,124], but other breeds develop atheromatous lesions in conduit arteries after feeding with high cholesterol diets [112,113,125,126]. Sex differences for some vascular functions and castration with hormone replacement indicate that both sex and hormones modulate these effects [127-129]. Rabbits also express the same myocardial contractile protein isoforms as humans (for example, β versus α myosin heavy chain in the adult ventricle) and have a myocardial blood reserve that more closely resembles humans [130] more so than that of rats or dogs [131]. Based on these similarities to humans, rabbits have also been used to evaluate acute myocardial infarction therapies [132-134].

Rabbits and rodents are coprophagic thus influencing dietary sources of protein [135]. It is unclear how this activity influences immunological responses in these species related to nitric oxide synthase Il (iNOS) or other isoforms of NOS that might be regulated by estrogen and are associated with inflammatory responses proposed as a stimulus for endothelial dysfunction and vascular disease [136-139].

Larger mammals, such as cattle, sheep, dogs, pigs and primates, offer the advantage of scale for testing devices and procedures to be developed in humans (for example, [140]) but are disadvantaged by availability, cost, and special requirements for handling and husbandry. While some large animals are available from commercial breeders, others are not, and it may be economical for some tissues to be obtained from abattoirs (cattle, pigs). However, limitations on sources may make it difficult to determine the sex and or hormonal status of the tissue donor. Commercially available animals may be castrated at birth, which affects hormonally mediated developmental processes, or may be sexually immature at the time of study making extrapolation of data to adult animals problematic. Retired breeder females have been used for studies of aging, but it is unclear if cardiovascular function(s) of multiparous animals are the same or different from age-matched nulliparous females or aged matched males. Reckelhoff and colleagues reported that renal function was decreased in Sprague Dawley female rats who had had six to seven pregnancies and lactations compared to virgin females [141], whereas Baylis and colleagues reported that renal function and blood pressure were not affected by three pregnancies and lactations in spontaneously hypertensive rats [142]. Retired breeder rabbits have been used for models of cell-based treatment for acute myocardial infarction and seem to be a reasonable model for human disease [132-134]. Thus, studies in retired breeders may more closely mimic the cardiovascular systems of most women than do virgin female animals.

Historically, dogs were the experimental animal of choice for investigating mechanisms of cardiac regulation and autonomic control of the vasculature, renal function, models of disease, development of imaging modalities, testing novel therapeutics, basic pharmacology of endothelial and smooth muscle function and aging [143-153]. Depending on the source of the animals, however, it may not always be possible to obtain information on age or reproductive history. While studies of ovariectomized and hormone replaced female dogs have been performed [152,154], studies in castrated male dogs are not reported in cardiovascular literature.

Swine smaller than 100 kg used in research are usually sexually immature, with the exception of Yucatan mini-swine. Depending on the supplier, males may be castrated at birth, so designation of 'male' with a weight of less than 100 kg may not represent results comparable to sexually mature animals [155-157]. Alternatively, sexually immature females (about 3 months of age) are used to maintain manageable sizes. The level of maturity or hormonal status for these animals is often not reported in methods sections of scientific papers. Ossabaw miniature swine (*Sus scrofa*) have a 'thrifty genotype' that when fed a high caloric diet enables them to store fat in order to survive seasonal food shortages. The phenotype of these female animals including central obesity, insulin resistance, impaired glucose tolerance, dyslipidemia and hypertension, are characteristics comparable to those used to define metabolic syndrome in humans. The atherosclerotic lesions in coronary arteries of the diabetic swine are similar to those found in humans and the size of the arteries are acceptable for testing coronary interventions such as stenting [158-160], thus providing an appropriate experimental animal to evaluate basic mechanisms of type II diabetes and treatment strategies which might be more easily be translated to human disease/treatment [159].

Non-human primates have been used extensively to study the natural history of atherosclerosis in relation to sex differences (See [77,161]). Rhesus and cynomolgus monkeys (*Macaca mulatta*, *Macaca fascicularis*, respectively) are particularly useful when fed diets that elevate blood lipids and the animals develop lesions consistent in morphological characteristics and location with those in humans with hyperlipidemia [78]. For example, atherosclerosis develops first in the aorta and proximal portions of the main branch coronary arteries and later in the common and internal carotid arteries. At risk animals (that is, those consuming an atherogenic diet) experience myocardial infarction at a rate similar to that of their human counterparts [162]. Finally, the macaques and other Old World anthropoid primates uniquely resemble women in reproductive function, as exemplified by similarities in ovarian hormone profiles, the presence of a menstrual cycle, and the occurrence of menopause [163].

An extensive series of studies, most conducted using socially housed female and male cynomolgus monkeys fed a diet relatively high in fat and cholesterol (designed to mimic typical consumption in industrialized countries) has resulted in seminal observations affecting design of future investigations with translation to human studies and evaluation of cardiovascular risk. The points below delineate these key findings.

(1) Females typically develop less atherosclerosis than males (see [77,164]). However, among socially housed monkeys this female 'protection' extends only to animals dominant within their social group; subordinate females exhibit a 'precocious acceleration' of atherosclerosis and are equivalent to males in coronary artery atherosclerosis extent.

(2) The precocious atherosclerosis that characterizes subordinate females likely results from a subclinical stress-induced, reversible ovarian impairment that resembles functional hypothalamic amenorrhea/anovulation (FHA) observed in women [165,166]. This hypothesis is supported by the observation that ovariectomy eliminates the protection of dominant females, rendering them equivalent to subordinate females and males in atherosclerosis extent [165,167]. Treatment of subordinate animals with exogenous estrogen (oral contraceptives) inhibits the development of atherosclerosis [168,169].

(3) The trajectory of premenopausal atherosclerosis predicts postmenopausal atherosclerosis extent. In a two-part study reproductively intact monkeys consumed an atherogenic diet for 2 years, half were also treated with an oral contraceptive [168]; all animals were ovariectomized and continued to consume an atherogenic diet for 3 more years. Atherosclerosis extent at ovariectomy (as determined in an iliac artery biopsy) predicted the amount of atherosclerosis present at the end of the study [169,170], irrespective of postmenopausal interventions.

(4) The extensive atherosclerosis that accompanies ovariectomy can be substantially inhibited by treatment with exogenous estrogens (for example, conjugated equine estrogens or 17β estradiol), only if treatment begins immediately following ovariectomy in animals initially free of atherosclerosis [171]; treatment of animals with pre-existing atherosclerosis is ineffective in proportion to the amount of lesion present [172,173].

The primary lesson from studies of female monkeys is that stress-induced premenopausal ovarian dysfunction, a common and subclinical condition, puts affected individuals on accelerated trajectory for atherosclerosis. Furthermore, the trajectory of atherosclerosis established in the premenopausal years appears to determine postmenopausal lesion extent, underscoring the importance of early events in the development of the postmenopausal disease burden.

Although studies in non-human primates have provided data for several decades of research and form the basis of studies in humans, the cost and difficulty of working with these animals make them prohibitive for many investigators in the future. In this regard, the US National Institutes of Health (NIH)-sponsored National Primate Research Center program and specialized colony resources (the Animal and Biological Materials Resources program) may provide investigators with access to animals and expertise to conduct studies with monkeys. Access to these resources is described at the website of the Division of Comparative Medicine (currently part of the National Center for Research Resources but soon to be moved into the Office of the NIH Director).

#### Establishing hormonal status in experimental animals

If hormones are implicated in the pathogenesis of cardiovascular disease, then it is incumbent upon those studying cardiovascular disease in experimental animals to understand how to quantify gonadal function. In experimental animals, frequency of estrus in females is species dependent: polyestrus with several cycles throughout the year (mice, rats, pigs, primates including humans), seasonal polyestrus with several cycles occurring at particular times of the year (sheep, hamsters) or diestrus with two cycles per year (dogs).

In order for female rats and mice to exhibit estrous cycling, they must be maintained in a 12 h light/dark cycle in a temperature-controlled room free of vibrations or external noise. Rats and mice of both sexes can be fertile as early as 5 to 6 weeks of age (37.3 ± 0.7 days [174]), although the age at puberty can vary in female mice depending on whether males are present. In rats and mice, the estrous cycle in adult females is 3 to 5 days, and gestation is 21 to 22 days. Animals caged together will often cycle together. In addition, rats and mice cease estrus cycling with aging (similar to menopause), but the age may vary with strain [175]. For example, Sprague Dawley rats stop estrous cycling at 16 to 18 months of age. However, female hypertensive rats, such as the spontaneously hypertensive rat and the Dahl salt sensitive rat maintained on a low salt diet (0.3% NaCl), both stop estrous cycling between 10 to 12 months of age [91,176]. At the beginning of estrous cycle cessation, vaginal smears show constant diestrus cells that lasts 1 to 3 months. Rats eventually cycle into constant estrus. Mice are similar and stop estrous cycling at 11 to 16 months of age depending on strain [175].

A common way to determine the progression through the cycle is by vaginal cytology. This method in mice is described in detail elsewhere [19] and is similar for rats. Briefly, wetted cotton swabs are placed into the vagina of the rat or mouse and twirled. Swabs containing accumulated cells are rolled onto dry glass slides and the slides are allowed to dry. The dry slides are stained with 0.1% toluidine blue, allowed to dry, and cell populations are viewed with a light microscope to observe cell morphology and populations. Alternatively, for vaginal lavage, 50 μl of deionized water is placed into the vaginal vault with a plastic pipette taking care not to stimulate the cervix [177] that can result in pseudopregnancy. (Just as in pregnant animals, pseudopregnant animals develop physiological changes, including weight gain, increases in glomerular filtration rate, renal plasma flow, and cardiac output, until days 11 to 13 when they revert back to the physiology of the non-pregnant state [178,179].) The pseudopregnant model is useful for studies of early pregnancy without the presence of the fetoplacental unit, and development of pseudopregnancy in rats and mice can also be accomplished by placing the females with vasectomized males.

On vaginal cell morphology, diestrus is characterized mainly by polymorphonuclear neutrophils (PMNs). Proestrus is characterized by a preponderance of larger nucleated epithelial cells. Estrus is characterized by irregularly shaped, cornified cells that contain pyknotic or no visible nuclei. Metaestrus is characterized by a combination of cornified cells and PMNs [19]. 17β-estradiol and progesterone levels are highest during proestrus and lowest during estrus [180-182]. To achieve pregnancy, female rats and mice are placed with a male preferably during diestrus. The presence of sperm on the vaginal smear or presence of a sperm plug is termed 'day 1 of pregnancy'.

Staging ovarian cycle in female rabbits is difficult because rabbits are in a prolonged state of proestrus. Vaginal probing initiates a surge in gonadotropins that induces ovulation making them similar to other species such as mink, ferrets, domestic cats, voles, and camels in being characterized as 'reflex ovulators'. In contrast, the surge in gonadotropins resulting in ovulation originates internally in rats, pigs, sheep, cows, non-human primates, and women, leading to their designation as 'spontaneous ovulators' [183]. Ovarian cycles in swine are monthly and females housed together will synchronize their cycles.

Staging reproductive cycle stages may also be discerned by visual (vulvar swelling or discharge), behavioral signs, vaginal impedance, steroid levels in urine or serum, or reproductive tract histology depending on species. It is difficult to identify estrus in female pigs by changes in external genitalia and estrus is often staged by behavioral postural changes in the presence of a male. Female swine are sensitive to stress, such as changes in housing, which can disrupt cyclicity. As discussed, anthropoid primates, including Old World monkeys and women, appear especially sensitive to the suppressive effects of negative social interaction or environmental circumstances [163,165,184].

Gonadal function is rarely quantified in studies using male animals except with identification of reproductive maturity and orchiectomy. In humans, men exhibit declines in testosterone and sperm production when stressed by metabolic/nutritional/energetic deficiencies and women exhibit declines in ovarian function and fertility when stressed, but what is stressful to women differs from what is stressful to men. Comparable studies in most experimental animals are lacking. In non-human primates, suppressed ovarian function secondary to social stress is common and is clearly associated with precocious acceleration of cardiovascular disease [77,161,184]. These effects of androgens have not been systematically assessed on cardiovascular disease of male monkeys, although atherosclerosis is exacerbated in ovariectomized females treated with either androstenedione or testosterone [185].

#### Delivery of hormones to castrated animals

Timing of castration/gonadectomy (that is, removing the gonads shortly after birth prior to sexual development or after puberty) in animals may be critical in the development or progression of cardiovascular disease. Caution should be exercised in the choice of hormones used to treat (replace) in a particular study. For example, both testosterone and 17β-estradiol are metabolized to biologically active substances. Progesterone at high concentrations binds to glucocorticoid receptors and some synthetic progestogens may have greater affinity for these receptors than others (see section 'What are sex steroid hormones?' above).

Sex steroids are typically given to experimental animals either by intramuscular injection or by subcutaneous implantation of time-release pellets. For intramuscular injections, an oil vehicle, such as castor oil or sesame oil is used. The disadvantage to this method is that daily injections are required for chronic studies. For studies in which sustained and consistent levels of steroids are the objective, commercially available pellets containing estradiol, progesterone, testosterone, dihydrotestosterone, or placebo, are implanted subcutaneously on the back of the animals. Pellets of different release times are available to achieve the plasma levels desired. Alternatively, sex steroids can be packed into medical grade silastic tubing, capped with silastic glue and implanted subcutaneously on the back of the animal [186]. The delivery dose depends on the length of the tube and the amount of steroid in the tube. These implants may be equilibrated by incubation in physiological saline for 24 hours to reduce the variability in initial release. With either of these subcutaneous delivery methods, plasma levels of hormones should be monitored for validation.

To mimic estrous cyclicity similar to normal rats and mice, ovariectomized animals can be injected with estradiol and progesterone, as described by Kramer and Bellinger [182]. These investigators used osmotic minipumps to deliver low doses of 17β-estradiol benzoate in ovariectomized female rats to mimic levels during the diestrus and estrus phases. Rats then received additional 17β-estradiol benzoate injections every 5 days to mimic proestrus increases. The investigators performed vaginal smears twice daily and found that the estrous cycles in the treated rats mimicked the cycles in intact females (for details on vaginal cytology, see [19]).

Oral preparations of sex steroids can be used but care is needed to assure that the animal consumes the steroid. Non-human primates can be easily trained to take measured amounts of oral formulations, however hormones can also be delivered to these species in the diet, for example, [169,187]. Final blood concentrations reached with given preparations (concentration) depends on steroid metabolism related to first pass metabolism in the liver.

#### Measurement of sex steroids

It is usually possible to obtain a blood sample for the measurement of sex steroids in experimental animals. The gold standard for measurement of sex steroids such as testosterone is liquid chromatography linked with mass spectroscopy [188]. This method removes any possibility of cross identity of antibodies with similar steroids, such as testosterone with 5α-dihydrotestosterone or estradiol, in ELISAs or radioimmunoassays (RIAs). This method may not be cost effective or feasible in all research or clinical environments.

Various ELISA or RIA kits for sex steroid measurement are commercially available. Estradiol and dihydrotestosterone are the most difficult to measure and often require extraction of plasma or serum to obtain consistent results. RIA kits may be difficult to use since the isotope used for many kits is radioactive iodine-125 and requires special precaution for use, radioactivity institutional permission, and disposal of gamma radioactivity.

One potential way to mitigate the problems associated with determination of circulating hormones is to assess such hormone concentrations in conjunction with measurement of menstrual cyclicity (as determined by vaginal swabbing). It has been observed in monkeys, for example, that abnormalities in ovarian hormone profiles are mirrored by irregularity in menstrual cyclicity [184].

One precautionary note is that measurement of hormones in the circulation may not be reflective of the active product at the tissue or cellular level because of the local metabolism of the sex steroids. Many organs outside of the reproductive organs contain receptors for sex steroids, and have the capacity to synthesize these steroids due to the presence of cytochrome P450 enzymes (see section 'What are sex steroid hormones?' above). For example, the kidney can produce sex steroids and androgen receptor and ERα are present in renal vascular and tubular cells [42]. However, the role that extragonadal steroid synthesis plays in mediating cardiovascular alterations in specific organs is not clear and requires further investigation.

The other compartment of interest may be the cerebrospinal fluid (CSF). Circulating levels and CSF levels of sex steroids have rarely been simultaneously quantified. In the systemic circulation buffering mechanisms keep hormonal excursions within a given range; these mechanisms do not seem to operate in the CSF. Hormones, especially cortisol, in CSF are not bound to any significant degree, thus, CSF hormone levels are 'free' and therefore bioactive [189]. These differences might be important in etiology of cardiovascular disease as much as cardiovascular regulation including that driven by stress and baroeceptors and chemoreceptors related to hypertension depends on neural mechanisms.

### Identifying genes that exhibit sex-specific or sex-interaction effects

An approach to the identification of genes that influence cardiovascular disease and related phenotypes involves genotyping a large number of individual progeny from a cross involving inbred (or in some circumstances outbred) strains of mice, rats, flies, or another model species, and using statistical tests to determine if there are loci that harbor genotypes or genetic variants that cosegregate with the phenotype of interest. This basic approach, often referred to as 'quantitative trait locus' (QTL) mapping has been pursued for years, with a number of published reviews describing relevant strategies for implementing the approach, successes with the approach as well as issues with its various formulations [190,191]. QTL mapping studies rarely, if ever, lead to the actual identification of phenotypically relevant causative genetic variants, but rather lead to the identification of a genomic region that is likely harboring a phenotypically relevant variant. This fact has important implications for QTL mapping studies seeking to identify genetic variants that contribute to sex-specific cardiovascular phenotypes, since, if evidence for cosegregation between a genetic variant and a sex-limited to sex-specific effect was found in QTL mapping study, work would still be required to identify the actual causal variant(s) and understand its molecular or gross physiologic impact or function.

Unfortunately, few QTL mapping studies have identified, or even attempted to implicate sex-dependent gene or genetic variant effects. However, QTL mapping strategies could easily accommodate testing hypotheses surrounding sex-dependent penetrance or sex × gene interaction effects, as described below. In addition, phenotypic comparisons across inbred (or outbred) strains are often pursued in anticipation of a QTL mapping study and could also easily accommodate sex-specific analyses. Results of such strain comparisons could motivate the search of genetic variants that have sex-limited or sex-dependent effects.

There is a rich established literature base describing the use of many different model species in genetic analyses (including QTL mapping studies) of cardiovascular disease, such as fruit flies [192,193] and baboons [194,195]. However, studies involving mice and rats have been pursued more frequently in strain comparison and QTL mapping studies. This prominence is more than likely due to the availability of many inbred mouse and rat strains that have been characterized for differences in cardiovascular phenotype profiles. The strain comparison and QTL mapping strategies and resources described below, all involving different mouse or rat strains, have been pursued by geneticists to identify genes contributing to cardiovascular disease related phenotypes that exhibit sex-specific effects.

#### Basic strain difference analysis

Many studies have investigated differences in cardiovascular disease-related phenotypes between different mouse and rat strains (see, for example, [196,197]). Since many mouse and rat strains have been inbred to homozygosity and have been reared in the same laboratory conditions, phenotypic differences between the resulting (clone-like) animals from different strains can be attributed to DNA sequence differences between them. Analyses of strain differences can easily accommodate sex differences if male and female animals with phenotype information are available. As simple in orientation as such studies may be, however, they have not been pursued with great frequency, probably because of the number of individual mice or rats one might need to detect realistic yet statistically significant strain × sex interaction effects that could be prohibitive. As an example of a strain comparison study involving cardiovascular phenotypes, Nadeau and colleagues contrasted homocysteine levels and related phenotypes across multiple mouse strains [198] and found a number of differences in a number of additional cardiovascular disease-related phenotypes obtained via elaborate phenotyping protocols [199]. The assessment of strain and sex differences between different mouse strains is greatly facilitated through searchable online strain phenotypic characteristic databases and simple push-button statistical analysis tools, such as those provided in the Mouse Phenome Database [200]http://www.jax.org/phenome.

#### *In silico *genetic analysis

The genomes of many mouse and rat strains have been sequenced, and analyses of these genomes have revealed substantial differences between them [201-203]. By contrasting and correlating the genetic differences between strains with phenotypic differences between those strains, one could potentially identify genetic variants that are associated with those phenotypes. The use of extant strain genetic and phenotypic data in this context is referred to as '*in silico*' QTL mapping since it involves summary and statistical analysis of existing data and not the generation of new data [204]. Statistical analyses involving *in silico *QTL mapping studies can easily accommodate hypothesis tests concerning sex-specific genetic effects, and can be greatly enhanced through the use of strain phenotype databases (for example, the Mouse Genome Database [205]) and online statistical analysis tools, such as those assembled within the Mouse Phenotype Association Database (MPAD; http://mouse.cs.ucla.edu/perl/mpad/pcont.pl).

#### Individual crosses and QTL mapping

As noted, the most widely used approach for identifying genetic factors that influence a particular phenotype involving model organisms is QTL mapping, in which crosses between different strains are exploited that differ phenotypically. QTL mapping strategies can easily accommodate hypotheses about sex-specificity of genetic variants, assuming a sufficient number of male and female progeny are available, through the use of appropriate statistical models that accommodate interaction terms, such as regression models where a phenotype is taken as a dependent variable and genotype is an independent or predictor variable. The inclusion of sex as an additional independent variable as well as a genetic variant × sex interaction term can be used to test hypothesis about sex specific genetic variant effects. Jacob and colleagues explored the sex-specific genetic effects on hypertension-related traits using very sophisticated pharmacologic and phenotyping protocols in crosses involving inbred rat strains [206,207]. Chromosome substitution strains, in which chromosomes from a strain with a particular phenotype are introgressed through breeding programs into a strain without that phenotype, have been used to identify the chromosomes carrying sex-specific genetic factors for cardiovascular disease-related traits in rats [208]. Nadeau and colleagues have pursued similar, very elegant, studies with mouse strains [209,210].

A number of other researchers have pursued QTL mapping studies that have explored sex-specific or sex × genetic variant interaction effects, but not necessarily for specific cardiovascular disease-related phenotypes but cardiovascular related risk factors. For example, sex × genetic variant effects influence fat mass and body size [211,212] in addition to the prevalence of sex-specific variant effects on a wide variety of complex phenotypic endpoints [213]. Expression levels of particular genes have been evaluated as a way of understanding the molecular physiologic impact of genetic variants that may ultimately impact phenotypic expression [214]. Other considerations include how to characterize sex specific genetic networks that impact phenotypic expression using QTL mapping studies [60,215]. In addition, studies of diet-induced bone loss using QTL mapping strategies have found evidence for sex-specific variant effects [216].

#### The large-scale multiple mouse strain Collaborative Cross

Since crosses involving two strains can only exploit the DNA sequence variation within those two strains and hence are limited with respect to the amount and possible interactions among many different sequence variants, the 'Collaborative Cross' (CC) was initiated by a number of prominent mouse geneticists [217,218]. The CC was designed to consider multiway crosses of eight founder mouse strains, resulting in greater genetically determined phenotypic diversity among the progeny than a typically two-way cross. In addition, based on calculations of the diversity and progeny size generated by the cross, the CC could have an ability to map QTLs at an approximately 1 megabase resolution, meaning that a causal variant might be very close to a marker associated with a trait if identified through the CC. The CC is considered a community resource and will be an ideal model system for the study of sex by gene interactions on cardiovascular disease and related phenotypes. A very recent study leveraging the CC for the study of genetic factors underlying energy balance traits showcases its potential [219].

### Statistical analysis

Essential to advancing the science and developing treatments/approaches to reduce disparities in healthcare between women and men, the sex of the experimental material (cultured cells, animals, humans) must be reported. While this information was once routinely given in Methods sections of scientific papers, a recent survey of the basic science literature related to brain and behavior found that about 75% of published papers in areas of physiology and pharmacology related to brain research did not report the sex of the animals [16,17,220] and only about 20% of papers published in cardiovascular journals with high impact factors reported the sex of cells studied in culture [18]. Since sex is a dichotomous variable, data should be analyzed by sex. This analysis is separate from incorporating sex in multivariate analysis of data. Negative or null data should be reported. Knowledge is to be gained from understanding in which systems/pathways or responses sex matters and to understand and compare differences between sexes for conditions in which one sex is most affected or most protected [221].

## Conclusions

The following conclusions can be reached (see also Table 1). (1) Sex is a variable in defining mechanisms responsible for cardiovascular structure and function in health and disease, and must be considered for all experimental designs and system choices (that is, cell culture, isolated tissues and whole animals, including genetically manipulated animals). (2) Phenotypes dictated by the sex chromosomes are modulated by sex steroid hormones; therefore, sex and hormonal status are critical in the design of experiments. (3) If differential experimental results occur in animals based purely on sex, much can be gained by studying the 'most affected' as well as the 'most protected' sex. (4) Studies in animals need to account for hormonal/gonadal status in both males and females (that is, intact, gonadectomized, sexually immature, hormone replete or replaced), as well as reproductive history of females. (5) Experiments that utilize both female and male animals should be sufficiently powered to detect sex difference if they exist or if not to provide confidence in a null finding, which should be reported. Analysis by sex includes sex as a dichotomous variable as well as a covariate. (6) As numerous studies have identified differential responses between sexes in etiology, consequences and treatment of cardiovascular disease, the idea that studies of fundamental mechanisms of cardiovascular physiology and disease can be discerned by the study of one sex and extrapolated to the other is scientifically invalid.

**Table 1:** Considerations for design of studies of sex and cardiovascular structure and function in health and disease

1. Concepts of sex differences must be integrated into research design.	4. The hormonal and gonadal state must be considered along with reproductive history in females.
2. Phenotypes are dictated by sex chromosomes and modulated by sex steroid hormones.	5. From a statistical standpoint, studies should be powered to detect sex differences.
3. A sex difference may be the simplest possible explanation for a particular experimental outcome.	6. Extrapolation of experimental outcomes from one sex to the other is invalid.

## Competing interests

This work was made possible by a grant to the Society for Women's Health Research (SWHR) ISIS Cardiovascular Network. SWHR is a national, non-profit organization headquartered in Washington, DC, USA. VMM served as President of the Organization for the Study of Sex Differences, an affiliate of SWHR, from 2010 to 2012.

## Authors' contributions

This paper represents a collaborative project of the ISIS Cardiovascular Network with each author providing expertise in her/his specialty such that the information is integrated throughout the entire work. VMM developed the conceptual framework, developed sections related to definitions, experimental animals, steroid signaling, impact and final editing; JRK provided expertise for overall conceptual framework, sections related to non-human primates, and steroid signaling; NJS provided sections related to genomics and experimental design; PO provided expertise regarding overall conceptual design, translational impact of work, and editing; SLB provided expertise on sections related to translation, hormone definitions and signaling and non-human primates, NKW provided expertise for overall conceptual framework and editing; LJS provided conceptual expertise related to translational application; RCW provided expertise on conceptual design, methods for animals studies and cultured cells, methods for delivery of hormones, and impact; MM oversaw administrative aspects of the work and editing; MS provided expertise on conceptual framework, definition of terms, methods for measurement of steroids and editing; DAT provided expertise for conceptual framework, design aspects related to experiments using cultured cells and progenitor cells; CNB provided expertise for conceptual framework, translation to human studies, definitions and editing; JFR provided expertise on overall conceptual framework, definitions, experiments related to rodents, delivery and measurement of hormones and editing. All authors approved the final version.
